# Genome Sequence of Serratia fonticola Strain S14, Isolated from the Mosquito Aedes triseriatus

**DOI:** 10.1128/MRA.00099-20

**Published:** 2020-04-23

**Authors:** Shicheng Chen, Edward D. Walker

**Affiliations:** a Department of Microbiology and Molecular Genetics, Michigan State University, East Lansing, Michigan, USA; b Department of Entomology, Michigan State University, East Lansing, Michigan, USA; Indiana University, Bloomington

## Abstract

The bacterium Serratia fonticola strain S14, isolated from the midgut of a female Aedes triseriatus mosquito, has a genome size of 6,176,978 bp. The genome includes genes responsible for acyl-homoserine lactone-mediated quorum sensing, enterobactin, and aerobactin.

## ANNOUNCEMENT

Serratia fonticola strain S14 (previously named strain MSU001) was isolated from a female Aedes triseriatus mosquito originating from a laboratory colony founded in 1988 from a woodlot on the campus of Michigan State University, cycled through 3 to 4 generations per year since then, and annually supplemented with wild mosquitoes from the same location (1). This mosquito species is an important vector of La Crosse encephalitis virus in the United States (2). For isolation of *S. fonticola* S14, the mosquito midgut was dissected using sterile tuberculin syringes and needles, followed by plating insect contents plus sterile saline on Luria-Bertani agar (BD, USA) at 28°C. *S. fonticola* strain S14 proved to be genetically manipulatable (3). Heterologous expression of genes encoding mosquitocidal toxins in bacteria naturally associated with mosquitoes in nature may be a powerful strategy for the biocontrol of vector mosquitoes and their associated diseases (4, 5). Serratia fonticola strain S14 persists as a commensal in the adult gut of its natural host, *Aedes triseriatus*, after having been ingested earlier by larvae but not in the unnatural host Anopheles stephensi (3); exploring the genomic features of *S. fonticola* strain S14 may further an understanding of the interactions between bacteria and their mosquito hosts (5).

Serratia fonticola strain S14 was cultured in Luria-Bertani agar (BD, USA) under aerobic conditions at 28°C, followed by genomic DNA extraction using a Wizard genomic DNA purification kit (Promega, CA, USA). Next-generation sequencing (NGS) was carried out at the Research Technology Support Facility (RTSF) at Michigan State University using Illumina MiSeq paired-end sequencing chemistry (2 × 250 bp) after library construction using the Illumina TruSeq Nano DNA library preparation kit. The insert size was estimated to be 745 bp. The raw reads (335,284,500 bp) were quality trimmed to eliminate adapter sequences and low-quality ends, using CLC Genomics Workbench v10.0 (Qiagen) with default settings. Genome assembly using the same software produced 274 contigs ranging in size from 204 to 399,119 bp. After further cleaning short sequences (<250 bp) in order to reduce the high redundancy, the assembled genome had 100 contigs with an *N*_50_ scaffold size of 127,836 bp (more than 50-fold coverage, 96% completeness accessed by CheckM v1.0.18; 6). The genome comprised 6,176,978 bp with a G+C content of 53.6%. Gene annotation was carried out by NCBI Prokaryotic Genome Automatic Annotation Pipeline (PGAAP, v4.10) (https://www.ncbi.nlm.nih.gov/genome/annotation_prok/). There were at least 74 RNA sequences, including 66 tRNAs, 4 rRNAs, and 4 noncoding RNAs (ncRNAs), in the genome.

Serratia fonticola strain S14 has many genes encoding acyl-homoserine lactone biosynthesis-related proteins, such as 3-hydroxyacyl-acyl carrier protein dehydratases FabA and FabZ, acyl carrier proteins (ACP1 and ACP2), and AHL synthases. In the S14 genome, 16 genes encoding enterobactin siderophore and 12 genes encoding aerobactin siderophore were found, indicating that this bacterium has a versatile ability to recover iron from the mosquito gut environment, where iron stress is important (7). These genomic data are important for us to understand the *S. fonticola* prevalence and persistence in specific mosquito species.

### Data availability.

The genome sequence of *S. fonticola* strain S14 was deposited in GenBank under accession number JAABOX000000000. The BioProject number for this project is PRJNA602295. The BioSample accession number is SAMN13887607. The raw sequence data are available under SRA accession number SRR10995863.
